# Patients treated by a medical trigger team at Sydvestjysk Sygehus Esbjerg

**DOI:** 10.1186/1757-7241-23-S1-A43

**Published:** 2015-07-16

**Authors:** Mikkel Brabrand, Lars Folkestad, Jacob B Brodersen

**Affiliations:** 1 Emergency Department, Sydvestjysk Sygehus, Esbjerg, Denmark; 2 Emergency Department, OUH Odense University Hospital, Odense, Denmark; 3 Institute of Regional Health Research, University of Southern Denmark, Odense, Denmark; 4 Department of Medicine, Sydvestjysk Sygehus, Esbjerg, Denmark

## Background

Dedicated teams receive patients in cardiac arrest and trauma patients at Danish Emergency Departments (ED). Until recently, there was no dedicated team for medical patients. However, as medical patients can be equally complex and critically ill, we found a need for an increased capacity to receive these patients. In March 2012, we therefore initiated a medical trigger team (MTT) for the sickest medical patients. Our MTT consist of two experienced physicians, two nurses, a laboratory technician and an orderly and the team is called when a patients meets certain criteria pre-hospitally. The team receives the patient in the ED and stays with the patient until stabilized or transferred to intensive care (ICU). The aim of this study was to present our initial findings.

## Methods

We retrieved a log of all calls of the MTT from 15 March 2013 until 28 May 2013 at Sydvestjysk Sygehus, Esbjerg. We identified patients treated by the MTT and extracted relevant information from the patient charts. We excluded children, patients transferred immediately to tertiary care, and cases where we could not identify the patients reliably. In this abstract, we present data on the demographics of these patients as well as their disposition and prognosis.

## Results

We had 103 activations of the MTT, two cases were unidentifiable, one was a child, one was cancelled and five patients were transferred immediately. We thus had 94 cases of which 46 (49%) were female and median age was 67 (range 15-96) years. Thirty-six (38%) patients presented with respiratory distress, 21 (22%) were unconscious, and 18 (19%) with convulsions. Median length of stay was 4 (range 0-23) days. Twenty patients (22%) were admitted directly to ICU and the rest to various medical departments. One patient (1%) died in the ED and one (1%) was immediately discharged. Seventeen (18%) died within one week, 24 (26%) within 30 days and 21 (22%) died in-hospital.

## Conclusions

Patients treated by our MTT have a high mortality and a large proportion is admitted directly to the ICU. It thus seems that our team is alerted for the right patients, i.e. the most critical medical patients.

